# Differentiating PC12 cells to evaluate neurite densities through live-cell imaging

**DOI:** 10.1016/j.xpro.2022.101993

**Published:** 2023-01-04

**Authors:** Jordyn Karliner, Diane E. Merry

**Affiliations:** 1 Department of Biochemistry and Molecular Biology, Sidney Kimmel Medical College, Thomas Jefferson University, Philadelphia, PA 19107, USA

**Keywords:** Cell Biology, Cell Differentiation, Microscopy, Neuroscience

## Abstract

Although PC12 cells are a valuable tool in neuroscience research, previously published PC12 cell differentiation techniques fail to consider the variability in differentiation rates between different PC12 cell strains and clonal variants. Here, we present a comprehensive protocol to differentiate PC12 cells into equivalent neurite densities through live-cell imaging for morphological, immunocytochemical, and biochemical analyses. We detail steps on optimized substrate coating, plating techniques, culture media, validation steps, and quantification techniques.

## Highlights

• Differentiate PC12 cells into neuronal cells with equivalent neurite densities
• Use live-cell imaging to track PC12 cell differentiation over time
• Quantify neurite densities to establish time point for secondary analyses
• Validate differentiation by Western blot and immunostaining for neuronal markers

## Abstract (editor highlights)

Publisher’s note: Undertaking any experimental protocol requires adherence to local institutional guidelines for laboratory safety and ethics.

## Graphical abstract

**Figure:**
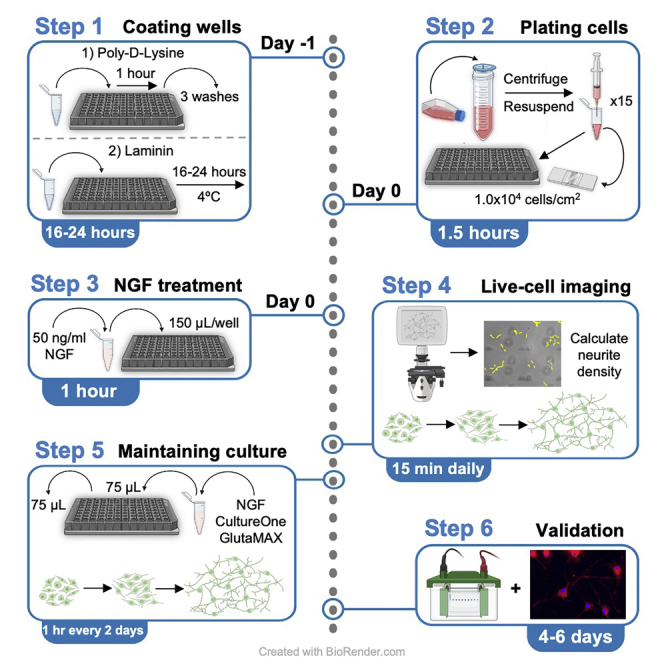

## Before you begin

**Figure 1:**
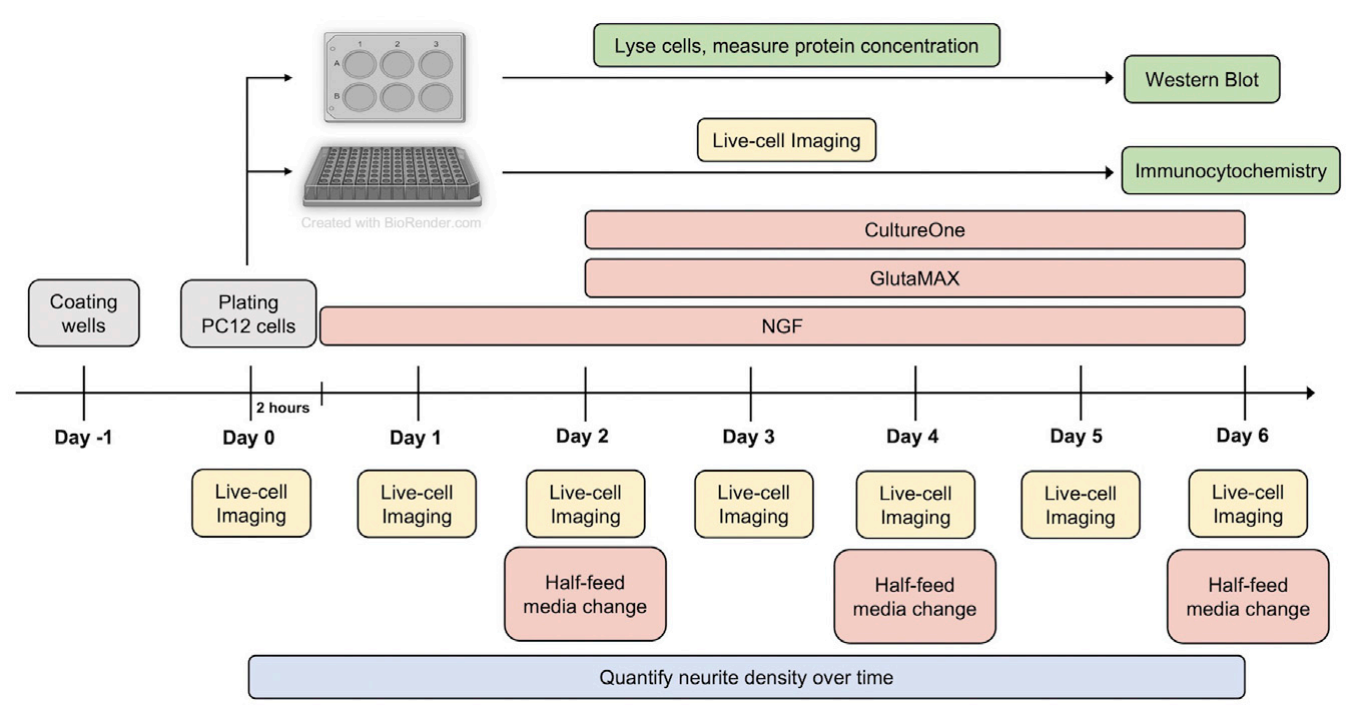
Schematic of experimental workflow for PC12 cell differentiation

This protocol provides detailed instructions to differentiate distinct PC12 cell strains and clonal variants to equivalent neurite densities through live-cell imaging, and validate the differentiation through western blot analysis and immunocytochemistry. PC12 cells are commercially available for purchase from a variety of companies including ATCC and Sigma Aldrich. This protocol utilizes a PC12 Tet-On® cell line from Clontech, which allows for inducible expression of any gene of interest under the control of a tetracycline-inducible promoter upon treatment with Doxycycline. Experiments included here were performed with PC12 cell clonal variants that contain inducible mutant forms of the androgen receptor^1^; for the data shown here, the androgen receptor was not expressed. Once differentiated, these neuronal cells can be used to evaluate genes, and mutant forms of these genes, involved in neurological function and disease, and to investigate their impact on neurite outgrowth and retention. For successful implementation of this protocol, researchers should have experience with techniques for general cell culture, microscopy, basic ImageJ/Fiji analysis, western blotting, and immunocytochemistry. See Figure 1 for a schematic of the experimental workflow. We recommend establishing the timing of differentiation for various PC12 cell strains and clonal variants prior to beginning validation studies. However, simultaneous plating of cells in 96-well plates for live-cell imaging and immunofluorescence, and 6-well plates for western blot analysis of neuronal markers, can be performed if desired.

### Prepare and aliquot nerve growth factor (NGF) stock solution

**Timing: 1–2 h**

1. Prepare 2 mL 0.1% Bovine Serum Albumin (BSA; stock = 7.5%) solution in Dulbecco’s phosphate-buffered saline (DPBS) without calcium or magnesium and filter through a 0.2 μm syringe filter into a sterile tube.
2. Reconstitute NGF (0.1 mg) in 1 mL 0.1% BSA solution (final concentration = 0.1 mg/mL).
3. Aliquot and store at −20°C for a maximum of 6 months.

**Figure 2:**
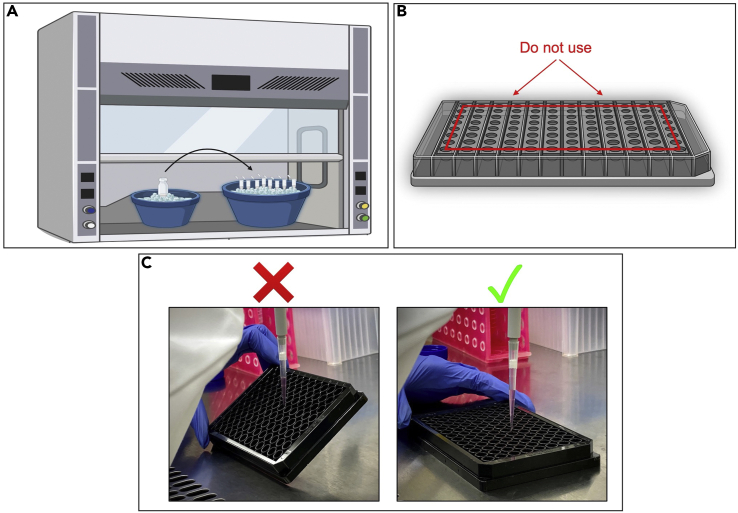
Critical steps for aliquoting reagents and plating PC12 cells for differentiation (A) Set-up for aliquoting NGF, laminin, and CultureOne in tissue culture hood. Use one or two ice buckets and keep reagent vials and all tubes on ice while aliquoting. (B) Wells to avoid using in a 96-well plate (red line) due to increased potential for evaporation in these wells. (C) Improper (left) and proper (right) technique for plating PC12 cells for differentiation. Cell culture plates should be flat on the hood surface and the pipette should be held vertical when adding cells. Graphics in A and B were created with BioRender.com.

**CRITICAL:** Prepare and aliquot NGF on ice in tissue culture hood (Figure 2A).

**CRITICAL:** Avoid freeze/thaw cycles. Aliquot in single-use 5, 10, and 25 μL aliquots.

### Aliquot laminin stock solution

**Timing: 1–2 h to thaw, 30 min to aliquot**

4. Thaw a vial of laminin at 4°C.
5. Once thawed, aliquot and store at −20°C for a maximum of 6 months.

**CRITICAL:** Aliquot laminin on ice in tissue culture hood (Figure 2A).

**CRITICAL:** Avoid freeze/thaw cycles. Aliquot in single-use 50–100 μL aliquots.

### Aliquot CultureOne supplement stock solution

**Timing: 5–10 min or 16–24 h to thaw, 30 min to aliquot**

6. Thaw a vial of CultureOne (100×) at 4°C for 16–24 h or at 37°C for 5–10 min or until just thawed.
7. Aliquot on ice in tissue culture hood (Figure 2A) and store at −20°C for a maximum of 6 months.

**CRITICAL:** Avoid freeze/thaw cycles. Aliquot in single-use 100–200 μL aliquots.

## Key resources table

**Table:** 

REAGENT or RESOURCE	SOURCE	IDENTIFIER
**Antibodies**		

Anti-beta III tubulin antibody (rabbit), 1:1000 for Western blot	Abcam	Cat#ab52623; RRID: AB_869991; Clone#EP1569Y
Anti-beta III tubulin antibody – neuronal marker (mouse), 1:1000 for immunocytochemistry	Abcam	Cat#ab78078; RRID: AB_2256751; Clone#2G10
GAP43 polyclonal antibody (rabbit), 1:500 for Western blot; 1:100 for immunocytochemistry	Invitrogen	Cat#PA1-24970; RRID: AB_794335
Synapsin-I antibody (rabbit), 1:1000 for Western blot and immunocytochemistry	Novus Biologicals	Cat#NB300-104; RRID: AB_10078308; Lot#ajo919p
Goat anti-mouse IgG (H+L) highly cross-adsorbed secondary antibody, Alexa Fluor 594, 1:500–1:1000	Invitrogen	Cat#A-11032; RRID: AB_2534091
Goat anti-rabbit IgG (H+L) highly cross-adsorbed secondary antibody, Alexa Fluor 488, 1:500	Invitrogen	Cat#A-11034; RRID: AB_2576217
Mouse anti-rabbit IgG-HRP, 1:2000	Santa Cruz Biotechnology	Cat#sc-2357; RRID: AB_628497

**Chemicals, peptides, and recombinant proteins**		

0.05% Trypsin EDTA (1×)	Gibco	Cat#25300-054
Bovine serum albumin solution (7.5%)	Sigma-Aldrich	Cat#A8412
Cell culture grade water	Corning	Cat#25-055-CVC
cOmplete™, Mini, EDTA-free Protease Inhibitor Cocktail (tablet)	Roche	Cat#04693159001
CultureOne™ Supplement (100×)	Gibco	Cat#A33202-01
Cytosine-beta-D-arabinofuranose hydrochloride (AraC)	Alfa Aesar	Cat#J65671
DMEM, 1× (Dulbecco’s Modification of Eagle’s Medium with 4.5 g/L glucose & L-glutamine without sodium pyruvate)	Corning	Cat#10-017-CV
Dulbecco’s phosphate-buffered saline (DPBS) with calcium and magnesium	Corning	Cat#21-030-CV
Dulbecco’s phosphate-buffered saline (DPBS) without calcium and magnesium	Corning	Cat#21-031-CM
Fetal bovine serum	R&D Systems	Cat#S11150
GlutaMAX™ Supplement, 200 mM (100×)	Gibco	Cat#35050-061
G418 Sulfate	Corning	Cat#61-234-RG
Hoechst 33342	Invitrogen	Cat#H1399
Horse serum	Gibco	Cat#16050122
Laminin Mouse Protein, Natural	Gibco	Cat#23017-015
L-Glutamine (200 mM)	Gibco	Cat#25030081
NGF-β from rat, recombinant, expressed in *Sf*21 cells	Sigma-Aldrich	Cat#N2513
Normal goat serum	Jackson ImmunoResearch	Cat#005-000-121
Opti-MEM^TM^ I Reduced Serum Medium (1×)	Gibco	Cat#31985-070
Penicillin-streptomycin (10,000 U/mL)	Gibco	Cat#15140122
Phenylmethylsulfonyl fluoride (PMSF)	Millipore Sigma	Cat#52332
Poly-D-lysine (0.1 mg/mL, molecular weight 50,000–150,000 daltons)	Gibco	Cat#A38904-01
Triton X-100	Fisher Scientific	Cat#BP151-500
Tween 20 (polysorbate 20)	Fisher Scientific	Cat#BP337-500

**Critical commercial assays**		

Clarity Western ECL Substrate	Bio-Rad	Cat#1705061
DC™ Protein Assay Kit II	Bio-Rad	Cat#5000112
TGX Stain-Free™ FastCast™ Acrylamide Kit, 10%	Bio-Rad	Cat#1610183

**Experimental models: Cell lines**		

PC12 Tet-On® Cell Line	Clontech (Takara)	Cat#630912; Available from the authors upon request

**Software and algorithms**		

Fiji	Schindelin et al.^2^	https://imagej.net/software/fiji/; RRID: SCR_002285

**Other**		

25 mm syringe filter 0.2 μm, Nylon, sterile	Fisher Scientific	Cat#09-719C
BD Syringe with Sub-Q Needle (1 mL, 26 gauge)	Fisher Scientific	Cat#14-829-10F
Biorupter® Pico sonication device	Diagenode	Previous: Cat#B01060010 Current: Cat#B01080010
ChemiDoc Imaging System	Bio-Rad	Cat#17001401
Corning® Sterile Cell Strainers (40 μm)	Corning	Cat#431750
EVOS™ M7000 Imaging System	Thermo Fisher Scientific	Cat#AMF7000
Immobilon®-P PVDF Membrane	Millipore Sigma	Cat#IPVH00010
Mini cell scrapers	Biotium	Cat#22003
Nunc MicroWell 96 well black/clear optical bottom plates	Thermo Fisher Scientific	Cat#165305
Nunc Poly-D-lysine coated 6 well plates	Thermo Fisher Scientific	Cat#152035

## Materials and equipment

**Table:** PC12 Cell Culture Medium

Reagent	Final concentration	Amount
Fetal Bovine Serum	5% (v/v)	25 mL
Horse Serum	10% (v/v)	50 mL
Penicillin-Streptomycin	100 U/mL	5 mL
L-Glutamine	4 mM	10 mL
G418 Sulfate^1^	100 μg	Varies based on starting concentration
DMEM (4.5 g/L glucose & glutamine without sodium pyruvate)	N/A	410 mL
**Total**	**N/A**	**500 mL**

Filter medium through a 0.2 μm filter unit. Store at 4°C for a maximum of 2 months.

^1^ Dissolve G418 Sulfate in DMEM and add to medium prior to filtering.

**Table:** PC12 Cell Stock Differentiation Medium

Reagent	Final concentration	Amount
Fetal Bovine Serum	2.5% (v/v)	625 μL
Horse Serum	5% (v/v)	1.25 mL
Penicillin-Streptomycin	100 U/mL	250 μL
GlutaMAX	4 mM	500 μL
Opti-MEM Reduced Serum Medium	N/A	22.38 mL
**Total**	**N/A**	**25 mL**

Filter medium through a 0.2 μm syringe filter. Store at 4°C for a maximum of 2 weeks. Make fresh medium for each experiment.

**Table:** 10× PBS

Reagent	Final concentration	Amount
NaCl	1.37 M	80 g
KCl	27 mM	2 g
Na_2_HPO_4_.7H_2_O	100 mM	27.2 g
KH_2_PO_4_	18 mM	2.4 g
ddH_2_O	N/A	Top to 1 L
**Total**	**N/A**	**1 L**

pH 7.2. Store at 20°C–25°C for a maximum of 2–3 months. If autoclaved, store for a maximum of 1 year.

**Table:** Triton DOC Lysis Buffer Stock Solution

Reagent	Final concentration	Amount
Sodium Deoxycholate	1% (w/v)	500 mg
10× PBS	10% (v/v)	5 mL
Triton X-100	0.5% (v/v)	250 μL
ddH_2_O	N/A	Top to 50 mL
**Total**	**N/A**	**50 mL**

Store at 20°C–25°C for a maximum of 3–6 months.

**Table:** 2× Sample Buffer Stock Solution

Reagent	Final concentration	Amount
20% Sodium Dodecyl Sulfate (SDS)	4% (v/v)	2 mL
Glycerol	20% (v/v)	2 mL
1 M Tris pH 6.8	125 mM	1.25 mL
Bromophenol Blue	0.004% (w/v)	0.4 mg
ddH_2_O	N/A	Top to 10 mL
**Total**	**N/A**	**10 mL**

Aliquot and store at 4°C for a maximum of 1 year.

**Table:** 10× Running Buffer Stock Solution

Reagent	Final concentration	Amount
Tris Base	250 mM	151.25 g
Glycine	1.92 M	721 g
ddH_2_O	N/A	Top to 5 L
**Total**	**N/A**	**5 L**

Store at 20°C–25°C for a maximum of 2–3 months.

**Table:** 1× Transfer Buffer

Reagent	Final concentration	Amount
Tris Base	47.88 mM	58 g
Glycine	386 mM	290 g
Methanol	20% (v/v)	2 L
ddH_2_O	N/A	Top to 10 L
**Total**	**N/A**	**10 L**

Store at 4°C for a maximum of 2–3 months.

**Table:** 10× Tris Buffered Saline (TBS) Stock Solution

Reagent	Final concentration	Amount
Tris Base	200 mM	24 g
NaCl	1.5 M	88 g
ddH_2_O	N/A	Top to 1 L
**Total**	**N/A**	**1 L**

Store at 20°C–25°C for a maximum of 2–3 months. If autoclaved, store for a maximum of 1 year.

**Table:** 1 × 0.05% TBST

Reagent	Final concentration	Amount
10× TBS	10% (v/v)	100 mL
Tween 20	0.05%	500 μL
ddH_2_O	N/A	900 mL
**Total**	**N/A**	**1 L**

Store at 20°C–25°C for a maximum of 2–3 months.

**Table:** 16% Paraformaldehyde

Reagent	Final concentration	Amount
Paraformaldehyde	16% (w/v)	8 g
ddH_2_O	N/A	50 mL
1 M NaOH	N/A	Dropwise
**Total**	**N/A**	**50 mL**

Heat paraformaldehyde and ddH_2_O to 60°C–65°C until milky, then add NaOH dropwise until solution clears. Filter through filter paper. Store at −20°C for a maximum of 1 year.

**CRITICAL:** Paraformaldehyde is flammable, may cause skin irritation and eye damage, and is harmful if swallowed or inhaled. Handle powder in fume hood and wear proper PPE. Dispose in proper waste container in fume hood.

***Alternatives:*** An alternative PC12 cell culture medium may be substituted for the one provided here if one is already in use by the researcher for general maintenance of cells.

***Alternatives:*** In addition to the Nunc MicroWell 96 Well Black/Clear Optical Bottom Plates used in this protocol, we have tested alternative plates that are suitable for PC12 cell differentiation. These include Corning® CellBIND® 96-well Flat Clear Bottom Black Polystyrene Microplates (Corning, Cat#3340) and Nunc MicroWell 96 Well Black/Clear Bottom Plate, Poly-D-Lysine (PDL) Coated Surface (Thermo Fisher Scientific, Cat#152037). If using pre-Poly-D-Lysine-coated plates, omit the PDL coating steps (coating wells steps 2–4) prior to laminin coating.

***Alternatives:*** This protocol uses the EVOS M7000 Imaging System equipped with an onstage incubator for live-cell imaging of differentiating PC12 cells. Other microscopes equipped with live-cell imaging capability may be suitable as well.

***Alternatives:*** This protocol was optimized using a validated tetracycline-inducible PC12 cell line from Clontech expressing high levels of the Tet-activator protein. However, this protocol is adaptable to various PC12 cell strains and clonal variants that may differentiate at different rates.

***Alternatives:*** 5× sample buffer (10% SDS, 50% glycerol, 312.5 mM Tris pH 6.8, 0.02% bromophenol blue, 25% 2-Mercaptoethanol (added at time of use)) may be prepared instead of 2× sample buffer.

***Alternatives:*** This protocol uses the Bioruptor® Pico sonication device for sonication of cell lysates, but an alternative sonicator may be used.

***Alternatives:*** This protocol uses the Bio-Rad DC™ Protein Assay Kit II to measure protein concentrations in cell lysates, but an alternative may be used.

***Alternatives:*** This protocol uses the ChemiDoc Imaging System for imaging total protein and membranes for western blot, but an alternative imaging system may be used.

## Step-by-step method details

### Coating wells—Day -1

**Timing: 16–24 h**

Wells in a 96-well plate are dual coated with PDL and laminin. PDL is used to promote cell adhesion, while laminin promotes attachment and differentiation of neuronal cells.

**CRITICAL:** Perform all steps under sterile conditions in tissue culture hood.

**CRITICAL:** To avoid evaporation of medium throughout the experiment, do not use the outermost wells of the plate (Figure 2B).

***Note:*** For initial differentiation, we recommend plating at least 3 wells per PC12 cell clonal variant to allow for quantification of neurite density during differentiation.

1. Sterilize wells in a 96-well plate under UV light in tissue culture hood for 30 min, with lid removed from the plate.
2. Prepare a 50 μg/mL working PDL solution by diluting PDL stock solution (0.1 mg/mL) in sterile DPBS without calcium or magnesium.
  a. Add 50 μL working PDL solution to each well.
  b. Incubate for 1 h at 20°C–25°C in tissue culture hood.
3. Wash each well 3 times with 100 μL cell culture grade water (sterile water for use in cell culture).

**CRITICAL:** Rinse wells thoroughly because excess PDL solution may be toxic to cells.

4. Remove last wash and let wells air dry in tissue culture hood for 2 h, with lid removed from the plate.
5. Prepare a 20 μg/mL working laminin solution by diluting laminin stock solution (concentration varies) in cell culture grade water.
  a. Add 100 μL working laminin solution to each well.
  b. Wrap the plate in parafilm and incubate for 16–24 h at 4°C.

**CRITICAL:** Thaw laminin aliquots on ice—laminin will inactivate if thawed at 20°C–25°C. Start thawing about 45 min prior to use. Prepare working laminin solution on ice (Figure 2A).

### Plating PC12 cells—Day 0

**Timing: 1.5 h**

PC12 cells are plated in a 96-well plate for NGF-induced differentiation.

**CRITICAL:** Perform all steps under sterile conditions in tissue culture hood.

***Note:*** Pre-warm all media to 37°C prior to use.

6. Remove 96-well plate from 4°C and incubate at 20°C–25°C in tissue culture hood for 1 h. During this 1-h incubation, PC12 cells are prepared for plating (steps 7–17).
7. Aspirate PC12 cell culture medium from PC12 cells (cultured in a T25 flask) and wash cells in 1 mL DPBS without calcium or magnesium.
8. Aspirate DPBS and add 1 mL 0.05% Trypsin EDTA.
  a. Incubate flask in a 37°C cell culture incubator with 5% CO_2_ for 2 min.
9. Add 1 mL PC12 cell culture medium to inactivate trypsin.
  a. Triturate cells 5–10 times with a P1000 pipette tip to thoroughly separate cells, while avoiding the formation of bubbles.

***Note:*** For this protocol, PC12 cells were cultured in a T25 flask prior to plating for differentiation. The above volumes may need to be adjusted if a larger volume flask or different culture vessel is used.

10. Pass cells through a 40 μm sterile cell strainer into a 50 mL sterile centrifuge tube.
11. Centrifuge cells for 3 min at 200 × *g* at 20°C–25°C.
12. Aspirate the liquid from the tube, leaving the cell pellet undisturbed.
13. Resuspend the cell pellet in 1 mL PC12 cell culture medium and transfer the PC12 cell suspension to a 1.5 mL sterile microcentrifuge tube.
14. Pass the PC12 cell suspension through a sterile 26-gauge syringe needle 15 times to create a single cell suspension (troubleshooting 1).

***Note:*** Work slowly to avoid forming bubbles.

15. Count cells on a hemocytometer.
  a. Create a 1:10 dilution of PC12 cell suspension by combining 10 μL PC12 cell suspension and 90 μL PC12 cell culture medium, and add 20 μL to the hemocytometer.
16. Calculate the volume of PC12 cells required per well to achieve a plating density of 1.0 × 10^4^ cells/cm^2^. Use the following formula, where “x” is the volume of cell suspension per well (mL):
  $$x=\frac{\left(Desired\;plating\;density\;\left(cells/cm^{2}\right)\right)\;\left(Growth\;area\;per\;well\;\left(cm^{2}\;\right)\right)\;}{Number\;of\;cells\;per\;mL\;\left(cells/mL\right)}$$
  a. For the 96-well plates used in this protocol, the growth area per well is 0.33 cm^2^.
17. Prepare diluted PC12 cell suspension for plating.
  a. Calculate the total volume of PC12 cell culture medium needed to plate a final volume of 100 μL per well (+ 3 extra wells) and aliquot into a new sterile tube.
  b. Multiply the calculated volume of cells per well (step 16) by the total number of wells being plated (+ 3 extra wells) to determine the volume of PC12 cell suspension to add to the aliquoted PC12 cell culture medium.
  c. From the aliquot of PC12 cell culture medium, remove and discard the volume calculated in step 17b and add the calculated volume of PC12 cell suspension to make the diluted PC12 cell suspension for plating.
    i. Pipette up and down a few times to mix.

***Note:*** Before preparing the diluted PC12 cell suspension, pipette the PC12 cell suspension up and down a few times to mix (troubleshooting 1).

18. Wash each well one at a time by aspirating the laminin and immediately adding 100 μL PC12 cell culture medium.

***Note:*** Working one well at a time ensures that the wells do not dry out, which is crucial to prevent laminin inactivation and cell detachment during culture.

***Note:*** If the 1-h 20°C–25°C incubation for the 96-well plate (step 6) is completed prior to completing steps 16 and 17, perform step 18 first.

19. Keeping the 96-well plate flat on the tissue culture hood surface, aspirate the medium from the wells one well at a time and add 100 μL diluted PC12 cell suspension to each well (Figure 2C).

**CRITICAL:** Do not let wells dry out while plating cells. Aspirate one well at a time and immediately add the diluted PC12 cell suspension.

***Note:*** Invert the diluted PC12 cell suspension after plating every 3–6 wells to prevent cells from settling to the bottom of the tube.

***Note:*** Pipette diluted PC12 cell suspension directly in the center of the well (Figure 2C) (troubleshooting 1).

20. Fill the unused surrounding wells in the 96-well plate with 200 μL DPBS.

**CRITICAL:** Do not tilt the cell culture plate while plating cells or adding DPBS to empty wells, as this will cause uneven distribution of cells throughout the well and may lead to cell clumping in one region (Figure 2C) (troubleshooting 1).

21. Carefully transfer the 96-well plate to a 37°C cell culture incubator with 5% CO_2_, avoiding tilting the plate to prevent uneven distribution of cells.

### Inducing neuronal differentiation—Day 0

**Timing: 1 h**

PC12 cells are treated with 50 ng/mL NGF to induce neuronal differentiation.

**CRITICAL:** Perform all steps under sterile conditions in tissue culture hood.

***Note:*** Wait at least 2 h after plating cells before beginning NGF treatment.

22. Prepare 25 mL stock Opti-MEM-based differentiation medium in a 50 mL sterile centrifuge tube according to the recipe provided here (adapted from Hu et al.^3^).
  a. Filter medium through a 0.2 μm syringe filter into a new 50 mL sterile centrifuge tube.
23. Prepare 50 ng/mL working NGF solution in stock differentiation medium.
  a. Calculate the total volume of stock differentiation medium needed for a final volume of 150 μL per well (+ 3 extra wells). Aliquot and pre-warm to 37°C.
  b. Calculate the volume of NGF stock (0.1 mg/mL) to add to the aliquoted stock differentiation medium for a final concentration of 50 ng/mL.
  c. From the pre-warmed aliquot of stock differentiation medium, remove and discard the volume calculated in step 23b and add the calculated volume of NGF stock.
    i. Pipette up and down a few times to mix.
24. One well at a time, remove the PC12 cell culture medium gently by pipette and discard, and add 150 μL working NGF solution.

**CRITICAL:** Do not let wells dry out while changing media. Change media one well at a time and immediately add working NGF solution.

***Note:*** Gently tilt the plate and put the pipette tip at the edge of the well to remove media. Do not aspirate media to avoid cell detachment. Slowly dispense media down the wall of the well to add. Do not add media directly into the well to avoid disrupting the cells.

25. Store cells in a 37°C, 5% CO_2_ cell culture incubator for the duration of cell culture.

### Live-cell imaging of differentiating PC12 cells—Day 0 and daily until end of experiment

**Timing: 15 min per day**

Differentiating PC12 cells undergo live-cell imaging daily to quantify neurite density over time.

***Note:*** The following steps describe live-cell imaging on an EVOS M7000 microscope. Set-up may vary with alternative microscopes.

***Note:*** If PC12 cells are imaged for longer than 20–30-min increments, use an onstage incubator set to 37°C, 5% CO_2_. If cells are imaged for longer than 1-h increments, also include humidity (80%).

26. Immediately after NGF treatment, image a set region of each well to track differentiation in the same fields of view over time. We recommend the following parameters:
  a. Use a 20×, short working distance objective.
  b. Use a transmitted light filter cube with phase contrast.
  c. Image 10% of each well from the center.
    ***Note:*** A different imaging area can be used, as long as this area remains consistent across time points and cell strains.
    ***Note:*** There is potential for a slight shift in the population of cells being captured each day due to shifts in the alignment of the plate in the vessel holder and cell migration, although this is very minimal. Capturing a set area of cells as opposed to selecting random fields of view throughout the well greatly reduces this risk and ensures the population of cells being analyzed remains consistent, since the impact of changes in cell location is minimal in a large area compared to a single field of view.
  d. Use autofocus set to “small structure” for optimal focus on the cells and neurites (troubleshooting 2).
  e. Save the data as a stitched tiled imaged to use for quantification of neurite density.
27. Repeat step 26 every 24 h to acquire images of the same fields of view to quantify neurite density over time.

**Figure 3:**
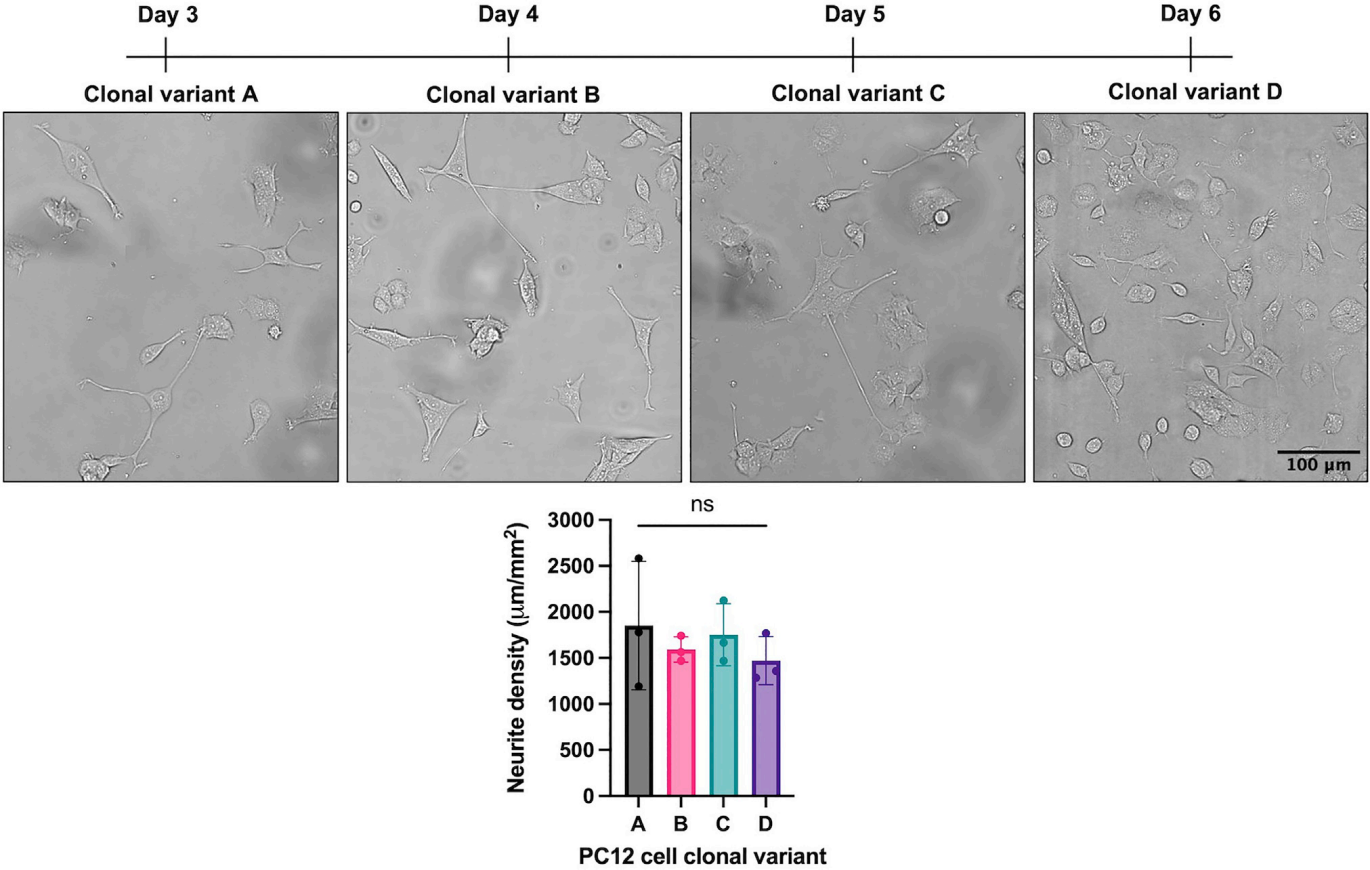
Different PC12 cell clonal variants vary in their differentiation rates Neurite densities of 4 different PC12 cell clonal variants each containing a different inducible mutant form of the androgen receptor (not expressed in these experiments), were quantified daily during differentiation until the culture reached a neurite density of ∼1,500 μm/mm^2^. The time it took for the 4 clonal variants to reach the target neurite density varied from 3–6 days of differentiation, and clonal variants also differed in their propensity to proliferate and clump during differentiation. Representative images of each clonal variant are shown on the day they reached the target neurite density. Three wells per clonal variant were analyzed and data represent mean ± SD with α < 0.05. Images are cropped for clarity.

**Figure 4:**
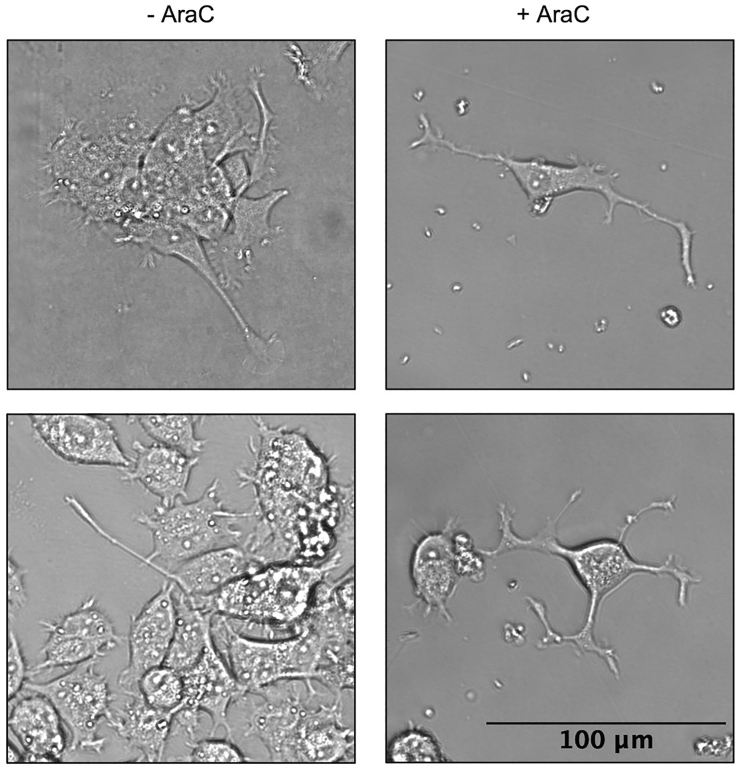
AraC treatment reduces proliferation and cell clumping in differentiated PC12 cells PC12 cells were differentiated to a neurite density of ∼1,500 μm/mm^2^ and treated with 1 μM AraC for 48 h. Three days after AraC treatment, neurite-bearing cells exist as single cells in culture while proliferating cells are greatly reduced. (right, + AraC). In contrast, at the same timepoint in the absence of AraC treatment, neurite-bearing cells exist in clumps (left, - AraC, top) or are overtaken by proliferating cells (left, - AraC, bottom). Images are cropped for clarity.

***Note:*** Once the total neurite density reaches ∼1,500 μm/mm^2^ in a 0.033 cm^2^ area (the total neurite length in 10% of one well), the cells are at a sufficient level of differentiation for secondary analyses (see quantifying neurite density over time). For the clonal variants used in this protocol, the desired total neurite density was reached in 3–6 days (Figure 3), although this window may be shorter or longer for other PC12 cell strains or clonal variants.

***Note:*** Neurites can continue to grow longer, and undifferentiated cells can grow new neurites, after the total neurite density in the well reaches ∼1,500 μm/mm^2^. Cells can be differentiated past this neurite density and used for secondary analyses, but Cytosine-beta-D-arabinofuranose hydrochloride (AraC) treatment may be needed to inhibit proliferation of undifferentiated cells and limit cell clumping (troubleshooting 3) (Figure 4). AraC interferes with DNA synthesis and therefore results in cell death of dividing cells without affecting neuronal cell survival or neurite outgrowth.^4^

### Maintaining differentiating PC12 cell culture—Day 2 and every 2 days until end of experiment

**Timing: 1 h every 2 days**

Differentiating PC12 cells are supplemented with NGF, CultureOne, and GlutaMAX every 2 days through half-feed media changes until PC12 cells reach a total neurite density of ∼1,500 μm/mm^2^. CultureOne is added to the medium to inhibit proliferation of undifferentiated cells during the differentiation process. AraC can also be used if there is an abundance of proliferation and/or cell clumping (troubleshooting 3) **(**Figure 4**)**.

**CRITICAL:** Perform all steps under sterile conditions in tissue culture hood.

28. Calculate the volumes of stock differentiation medium, NGF, CultureOne, and GlutaMAX needed for a half-feed media change. Assuming the reagents remaining in the media in the well are inactive/degraded, make up supplemented media with reagents at double the desired final concentration.
  a. Calculate the total volume of stock differentiation medium needed for a final volume of 75 μL per well (+ 3 extra wells). Aliquot and pre-warm to 37°C.
  b. Calculate the volume of NGF stock to add to the aliquoted stock differentiation medium for a working concentration of 100 ng/mL (final concentration in each well will be 50 ng/mL).
  c. Calculate the volume of CultureOne supplement to add to the aliquoted stock differentiation medium for a working dilution of 1:25 (final dilution in each well will be 1:50).
  d. Calculate the volume of GlutaMAX to add to the aliquoted stock differentiation medium for a working concentration of 4 mM (since there is already 4 mM GlutaMAX in the stock differentiation medium, the final concentration in each well will be 4 mM).
29. From the pre-warmed aliquot of stock differentiation medium, remove and discard the volumes calculated in steps 28b–d and add the calculated volumes of NGF, CultureOne, and GlutaMAX.
  a. Pipette up and down a few times to mix.
30. Remove and discard 75 μL of media from each well and add 75 μL stock differentiation medium supplemented with NGF, CultureOne, and GlutaMAX.
31. Repeat steps 28–30 every two days until the neurite density reaches ∼1,500 μm/mm^2^ (see quantifying neurite density over time).

### Validating neuronal differentiation—Day 0 through end of validation

**Timing: 4–6 days, in addition to cell culture time**

Differentiated PC12 cells should express the neuronal proteins β-III-Tubulin, Synapsin-I, and GAP43.^3^^,^^5^^,^^6^ To verify expression of these proteins, differentiating PC12 cells are lysed throughout the differentiation process and neuronal proteins are detected through western blot analysis. Once PC12 cells have differentiated to a total neurite density of ∼1,500 μm/mm^2^, cells are fixed and evaluated for expression of the neuronal markers through immunocytochemistry.

### Western blot analysis

**Timing: 2–3 days, in addition to cell culture time**

PC12 cells are plated on PDL-coated 6-well plates for differentiation. Cell lysates are collected for western blot analysis of neuronal proteins on Day 0 and every subsequent 2 days until the total neurite density reaches ∼1,500 μm/mm^2^ (as determined through steps 26 and 27 and quantifying neurite density over time).

***Note:*** Depending on the differentiation rate of the cells, lysates may need to be collected every day to observe the increase in neuronal proteins over time.

#### Coating wells

**Timing: 1 h**

32. Sterilize wells in 6-well plates under UV light in tissue culture hood for 30 min, with lids removed from the plates.
  a. Cells will be plated in triplicate for each timepoint at which cell lysates are collected. For example, if collecting lysates on days 0, 2, 4, and 6, a total of 12 wells will be required for the experiment.
  b. Cells to be lysed on different days need to be plated in separate 6-well plates. Therefore, the above example requires 4 plates.
  c. Label each plate with the day that cells will be lysed (i.e., “Day 0”, “Day 2”, “Day 4”, or “Day 6”).
33. Prepare a 20 μg/mL working laminin solution by diluting laminin stock solution in cell culture grade water.
  a. Add 1 mL working laminin solution to each well.
  b. Wrap plates in parafilm and incubate for 16–24 h at 4°C.

***Note:*** Although not tested in this protocol, alternative 6-well plates that are not pre-coated with PDL might be suitable for differentiation, but wells must be coated with PDL (1.5 mL 50 μg/mL PDL per well) prior to laminin coating.

#### Plating cells

**Timing: 1.5 h**

34. Follow steps 6–21 to plate PC12 cells, with the following alterations:
  a. In step 16, plate PC12 cells at a density of 1.5 × 10^4^ cells/cm^2^. This will yield a high enough protein concentration in the cell lysates to detect neuronal proteins through western blot.
  b. In step 17, a final volume of 2 mL will be added to each well of a 6-well plate.
  c. In step 18, add 1 mL PC12 cell culture medium to each well.
  d. In step 19, add 2 mL diluted PC12 cell suspension to each well.
  e. In step 20, fill the unused surrounding wells with 2 mL DPBS.

#### Inducing neuronal differentiation

**Timing: 1 h**

35. Follow steps 22–25 to induce neuronal differentiation for all plates except “Day 0” plate, with the following alterations:
  a. In step 22, prepare at least 50 mL stock differentiation medium (this volume will vary based on the number of wells being plated, so calculate the exact volume needed for the duration of the experiment before preparing media).
  b. In steps 23 and 24, a final volume of 2 mL will be added to each well.

#### Lysing cells, sonicating cell lysates, and measuring protein concentration

**Timing: 3 h**

36. Lyse cells in “Day 0” plate.
  **CRITICAL:** The cell culture plate and all solutions must be kept on ice while lysing cells. These steps do *not* need to be performed under sterile conditions.
  a. Prepare 3.5 mL working Triton DOC lysis buffer from Triton DOC lysis buffer stock solution by adding the following reagents:
    i. 17.5 μL 1 M MgCl_2_.
    ii. 7 μL 0.5 M EDTA.
    iii. ½ Mini, EDTA-free Protease Inhibitor Cocktail tablet.
    iv. 8.75 μL saturated Phenylmethylsulfonyl fluoride (PMSF) solution in isopropanol.
      **CRITICAL:** PMSF is toxic if swallowed and can cause skin corrosion. Wear proper PPE when handling.
      ***Note:*** Add PMSF to lysis buffer immediately before lysing cells.
      ***Note:*** Although not tested in this protocol, an alternative lysis buffer may be used for cell lysis.
  b. Aspirate media from the wells and wash cells in 1 mL DPBS with calcium and magnesium.
  c. Aspirate DPBS and add 30 μL working Triton DOC lysis buffer to each well.
    ***Note:*** Make sure all DPBS is aspirated from the well before adding lysis buffer to avoid diluting the cell lysates (troubleshooting 4).
  d. Scrape the cells in the well thoroughly in multiple directions with a mini cell scraper and add the cell lysate to a tube for sonication.
  e. Centrifuge lysates for 2–5 s in a mini centrifuge.

37. Sonicate cell lysates using a sonicator. In this protocol, the Bioruptor® Pico sonication device was set to 4°C and sonication settings were 6 cycles, 30 s on, 30 s off.

**Pause point:** Lysates can be stored at −80°C.

38. Measure protein concentrations in cell lysates using the Bio-Rad DC Protein Assay Kit II or other suitable alternative, according to standard protocol.
39. Store lysates at −80°C until all cells are lysed on subsequent days before running western blot.

***Optional:*** Lysates can be aliquoted prior to storing at −80°C to avoid excess freeze/thaw cycles if multiple western blots need to be run. Make aliquots in triplicate of at least 15 μg protein for each sample.

40. Repeat steps 36–39 every 2 days to lyse cells at multiple time points during the differentiation process.

**Figure 5:**
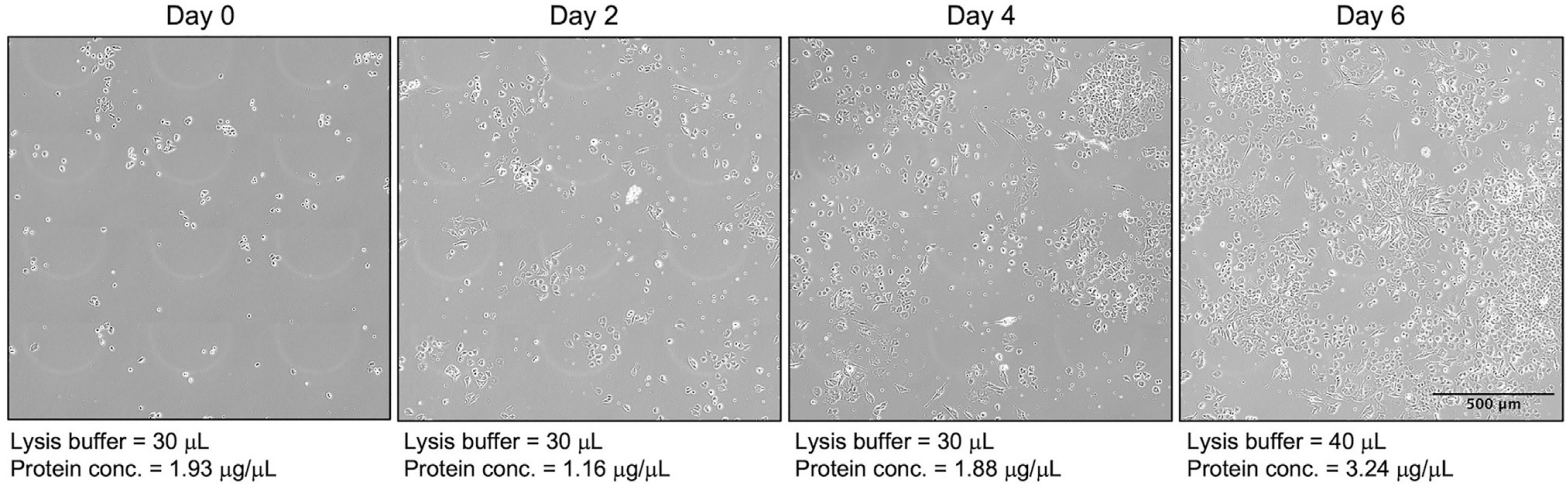
Cell confluency over 6 days of differentiation for western blot analysis Images depicting the confluency of PC12 cells every 2 days of differentiation, with corresponding lysis buffer volume used for cell lysis and average protein concentration in cell lysates. Images are cropped for clarity.

***Note:*** The volume of lysis buffer added to each well will likely need to be increased for lysis of cells on later days of the differentiation process to account for the increase in cell density/neurite outgrowth over time. See Figure 5 for details on cell confluency and corresponding lysis buffer volume and protein concentration (troubleshooting 4).

#### Maintaining differentiating PC12 cell culture

**Timing: 1 h every 2 days**

41. Follow steps 28–30 every 2 days to maintain the culture of differentiating PC12 cells through half-feed media changes with the following alterations:
  a. In step 28, a final volume of 1 mL per well is needed.
  b. In step 30, remove and discard 1 mL of media from each well and add 1 mL stock differentiation medium supplemented with NGF, CultureOne, and GlutaMAX.

***Note:*** Cells to be lysed on a specific day do not need to undergo a half-feed media change on that day. Therefore, every subsequent 2 days of the protocol, one fewer plate of cells will undergo a half-feed media change. Recalculate the volumes of media, NGF, CultureOne, and GlutaMAX needed for cell culture every 2 days to avoid wasting reagents.

42. Observe differentiating PC12 cells under an inverted light microscope every day to verify that the cells are differentiating as expected.

#### SDS polyacrylamide gel electrophoresis (SDS-PAGE), protein transfer, and immunoblotting

**Timing: 2–3 days (beginning after all cells are lysed)**

43. Prepare lysates for SDS-PAGE.
  **CRITICAL:** Keep lysates on ice until boiling (step 43e).
  a. Remove lysates from −80°C and thaw on ice.
  b. To an aliquot of 2× sample buffer stock solution, add 10% 2-Mercaptoethanol.
    ***Note:*** 2× sample buffer + 2-Mercaptoethanol can be stored at −20°C for a maximum of 1 month.
    **CRITICAL:** 2-Mercaptoethanol is toxic if swallowed or inhaled, causes skin irritation and corrosion, and may be fatal in contact with skin. It is also toxic to aquatic life. Use in fume hood and wear proper PPE while handling. Dispose in proper waste container in fume hood.
  c. Prepare 1× running buffer from 10× running buffer stock solution. Add 0.1% SDS to the 1× running buffer.
  d. If not already done, aliquot at least 15 μg of protein from each cell lysate sample, preparing triplicate aliquots of each sample in order to run 3 gels.
    i. To these aliquots, add an appropriate volume of 2× sample buffer + 2-Mercaptoethanol to the lysates for a final concentration of 1× and vortex each sample.
  e. Boil samples at 90°C–100°C for 5 min.
  f. Vortex samples and centrifuge for 5 s in a mini centrifuge.
  g. Load identical samples into 3 SDS-PAGE stain-free gels and run at 120 V in 1× running buffer + SDS.
    ***Note:*** Running three separate gels with identical samples allows detection of the three differentiation markers Synapsin-1, β-III-Tubulin, and GAP43 at the same time. Alternatively, two gels can be run, and after protein transfer and immunoblotting, one membrane can be stripped of bound antibody and reprobed for the third protein.
    ***Optional:*** If desired, PC12 (NGF-differentiated) lysates (ECM Biosciences Cat#PL7141) can be loaded on the gel as a positive control.
    ***Note:*** This protocol uses stain-free gels (poured using TGX Stain-Free™ FastCast™ Acrylamide Kit, 10%) for SDS-PAGE to normalize neuronal proteins of interest to total protein in each well. We have not evaluated whether expression of housekeeping genes used for normalization change with differentiation and therefore cannot guarantee their accuracy as a measure of total protein.

44. After the dye front has left the gels, image gels for total protein using a ChemiDoc or other compatible imaging system.
45. Transfer proteins from the gels to 0.45 μm polyvinylidene difluoride (PVDF) membranes in 1× transfer buffer according to standard protocol.
46. Incubate the membranes with the following blocking solutions for 1 h at 20°C–25°C:
  a. Membranes 1 and 2: 5% nonfat dry milk in 1 × 0.05% TBST (these membranes will be probed for Synapsin-1 and GAP43).
  b. Membrane 3: 5% BSA in 1 × 0.05% TBST (this membrane will be probed for β-III-Tubulin).
47. Incubate the membranes with the following primary antibodies diluted in their respective blocking solutions for 16–24 h at 4°C.
  a. Membrane 1: Synapsin-1 (Rb, 1:1000).
  b. Membrane 2: GAP43 (Rb, 1:500).
  c. Membrane 3: β-III-Tubulin (Rb, 1:1000).
48. Wash membranes 3 times for 15 min in 1 × 0.05% TBST.
49. Incubate membranes in anti-Rb HRP secondary antibody (1:2000) diluted in their respective blocking solutions for 1 h at 20°C–25°C.
50. Wash membranes 2 times for 15 min in 1 × 0.05% TBST.
51. Wash membranes 1 time for 15 min in 1× TBS.
52. Develop membranes with Clarity Western ECL Substrate using a ChemiDoc or other compatible imaging system.
53. Quantify total protein lanes and protein bands using Image Lab software or other quantification software. Normalize protein bands to total protein for each well.

### Immunocytochemistry

**Timing: 2–3 days**

Following differentiation of PC12 cells to a neurite density of ∼1,500 μm/mm^2^ (as determined in steps 26 and 27 and quantifying neurite density over time), cells are fixed and immunostained for Synapsin-1, GAP43, and β-III-Tubulin directly in the 96-well plate used for differentiation.

**CRITICAL:** All liquid should be removed from the wells gently by pipette to avoid loss of cells and neurites during the staining process (troubleshooting 5). Remove and add liquid 3 wells at a time to prevent cells from drying out.

**CRITICAL:** Secondary antibodies and Hoechst dye are light-sensitive. Protect the plate from light once they are in use.

***Note:*** Immunocytochemistry is performed after completing steps 1–31. These steps do *not* need to be performed under sterile conditions.

***Note:*** Although not tested in this protocol, cells may be plated on coverslips for differentiation and subsequent immunocytochemistry.

54. Fix cells in paraformaldehyde (PFA).
  a. Prepare 4% PFA in DPBS with calcium and magnesium from a 16% stock solution.
  b. Remove and discard 75 μL media from each well and add 75 μL 4% PFA (final concentration = 2% PFA).
    i. Incubate for 10 min at 20°C–25°C.
  c. Remove and discard all liquid from the wells and add 100 μL 4% PFA.
    i. Incubate for 10 min at 20°C–25°C.
  d. Remove and discard 4% PFA and wash cells 1 time in DPBS with calcium and magnesium.

**Pause point:** Plate can be wrapped in parafilm and stored at 4°C for up to 2 weeks before staining.

55. Prepare permeabilization and blocking solution of 5% normal goat serum (NGS) + 0.3% Triton X-100 in DPBS with calcium and magnesium.
  a. Remove and discard DPBS wash from the wells and add 100 μL NGS/Triton X-100 solution per well.
  b. Incubate for 1 h at 20°C–25°C.
56. Prepare primary antibody solutions in DPBS with calcium and magnesium + 5% NGS.
  a. Half of the wells will be incubated with β-III-Tubulin (Ms, 1:1000) and Synapsin-1 (Rb, 1:1000) primary antibodies.
  b. The other half of the wells will be incubated with β-III-Tubulin (Ms, 1:1000) and GAP43 (Rb, 1:100) primary antibodies.
  c. Remove and discard the permeabilization/blocking solution and add 80 μL of the appropriate primary antibody solution to each well.
  d. Wrap the plate in parafilm and incubate for 16–24 h at 4°C.
57. Wash cells 3 times for 10 min in 100 μL DPBS with calcium and magnesium.
  a. Put the plate on an orbital shaker at the lowest speed during washes (troubleshooting 5).
58. Prepare secondary antibody solution in DPBS with calcium and magnesium + 5% NGS.
  a. All wells will be incubated with Goat anti-Mouse IgG Highly Cross-Adsorbed, Alexa Fluor 594 (1:500–1:1000) and Goat anti-Rabbit IgG Highly Cross-Adsorbed, Alexa Fluor 488 (1:500) secondary antibodies.
  b. Remove and discard the DPBS wash from the wells and add 80 μL secondary antibody solution to each well.
  c. Wrap the plate in aluminum foil to protect from light and incubate for 1 h at 20°C–25°C.
59. Wash cells 3 times for 10 min in 100 μL DPBS with calcium and magnesium.
  a. Wrap the plate in aluminum foil to protect from light and incubate on an orbital shaker at the lowest speed during washes (troubleshooting 5).
60. To stain nuclei, prepare 1:500 Hoechst dye solution in DPBS with calcium and magnesium + 5% NGS from a 1 mg/mL working stock solution.
  a. Remove and discard DPBS wash from the wells and add 80 μL Hoechst solution to each well.
  b. Wrap the plate in aluminum foil to protect from light and incubate for 20 min at 20°C–25°C.
61. Remove and discard the Hoechst solution and add 100 μL DPBS with calcium and magnesium to each well.

**Pause point:** Before imaging, the plate can be wrapped in aluminum foil and parafilm and stored at 4°C for up to 2 weeks.

62. Image neurite-bearing cells with a fluorescence microscope equipped with a 96-well plate holder. We recommend using a 20× or 40× short working distance objective.

## Expected outcomes

**Figure 6:**
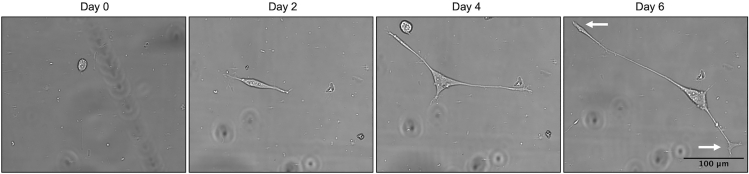
NGF-induced neurite outgrowth of PC12 cells over 6 days of differentiation Upon NGF treatment, PC12 cells undergo a morphology change from a circular to triangular-shaped cell body and gradually extend neurites that develop bulbous terminal ends (arrows). Images are cropped for clarity.

Using this protocol, researchers will be able to differentiate PC12 cells into neurite-bearing cells that express the neuronal markers Synapsin-1, GAP43, and β-III-Tubulin. These cells can be used to evaluate the role of proteins in neurological function and disease, and to investigate their impact on neurite outgrowth and retention. As PC12 cells differentiate, they undergo a change in cell body shape from circular to triangular and gradually extend neurites that develop bulbous terminal ends (Figure 6, arrows). This protocol details how to differentiate varied PC12 cell strains and clonal variants to equivalent stages of differentiation through live-cell imaging, and quantify neurite density over time. We show that 4 different PC12 cell clonal variants, each containing a different inducible mutant form of the androgen receptor (not expressed in these experiments), required between 3–6 days of differentiation before reaching the target neurite density of ∼1,500 μm/mm^2^, representative of the variability in differentiation rates between clonal variants. These different clonal variants also varied in the propensity of cells to clump together and proliferate, which may also impact time to differentiate (Figure 3).

**Figure 7:**
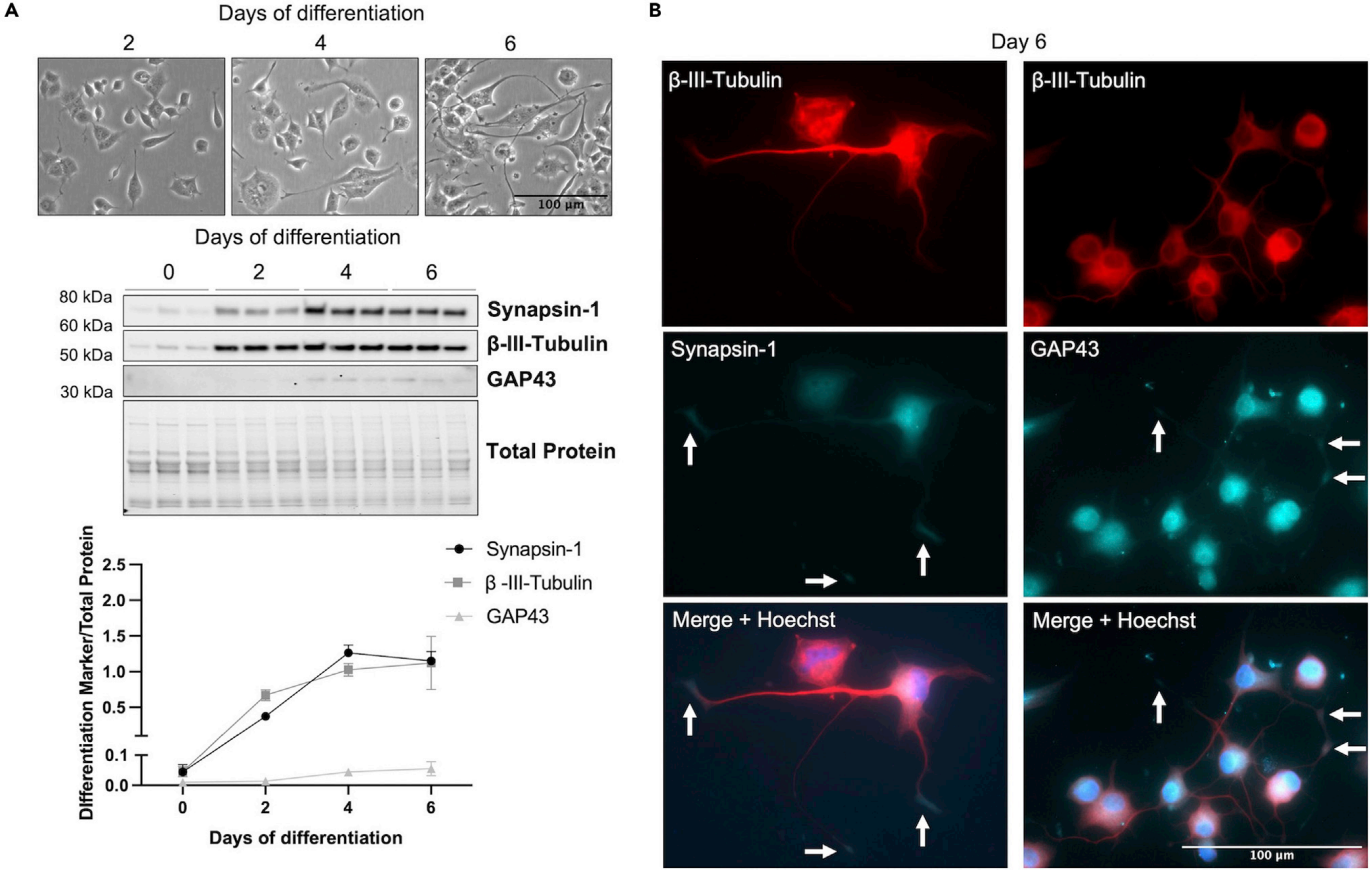
Differentiated PC12 cells express the neuronal markers Synapsin-1, β-III-Tubulin, and GAP43 (A) Expression of neuronal proteins Synapsin-1, β-III-Tubulin, and GAP43 increase over 6 days of differentiation, corresponding to neurite outgrowth. Phase-contrast images are cropped for clarity. (B) At 6 days of differentiation, neuronal proteins are visualized through immunofluorescence (β-III-Tubulin = red; Synapsin-1 and GAP43 = cyan (pseudo-colored)). Proteins are localized in the nucleus (Synapsin-1 and GAP43), cytoplasm (all), and neurites (all). Arrows indicate expression of Synapsin-1 and GAP43 in the bulbous terminal ends of the neurites. Images are cropped for clarity.

Expression of Synapsin-1, GAP43, and β-III-Tubulin should increase over time of differentiation.^3^^,^^5^^,^^6^ We show that these neuronal proteins are absent or expressed at very low levels in undifferentiated PC12 cells and their expression increases over time of differentiation, mirroring the extension of neurites (Figure 7A). As visualized through immunofluorescence on the final day of differentiation, β-III-Tubulin (red) is localized to the cytoplasm and neurites, while Synapsin-I and GAP43 (cyan, pseudo-colored) are localized to the nucleus, cytoplasm, and neurites, with prominent expression in the terminal ends of the neurites (Figure 7B, arrows). While not evaluated in this protocol, NGF-differentiated PC12 cells have been shown to express additional neuronal proteins, such as neurofilaments.^6^^,^^7^

## Quantification and statistical analysis

### Quantifying neurite density over time

**Timing: 15–30 min per image**

Live-cell images acquired daily are analyzed in Fiji^2^ to quantify the total neurite density in the same fields of view over time. It is best to analyze images on the day they are captured to determine whether continued differentiation is required to reach the target neurite density of ∼1,500 μm/mm^2^.

1. Starting with images acquired on day 1 of differentiation, open a tiled image file in Fiji (Figure 8A).
  a. Tiled images from the EVOS M7000 are displayed in inches. Neurite lengths will be converted from inches to μm after neurite tracing is complete.

**Figure 8:**
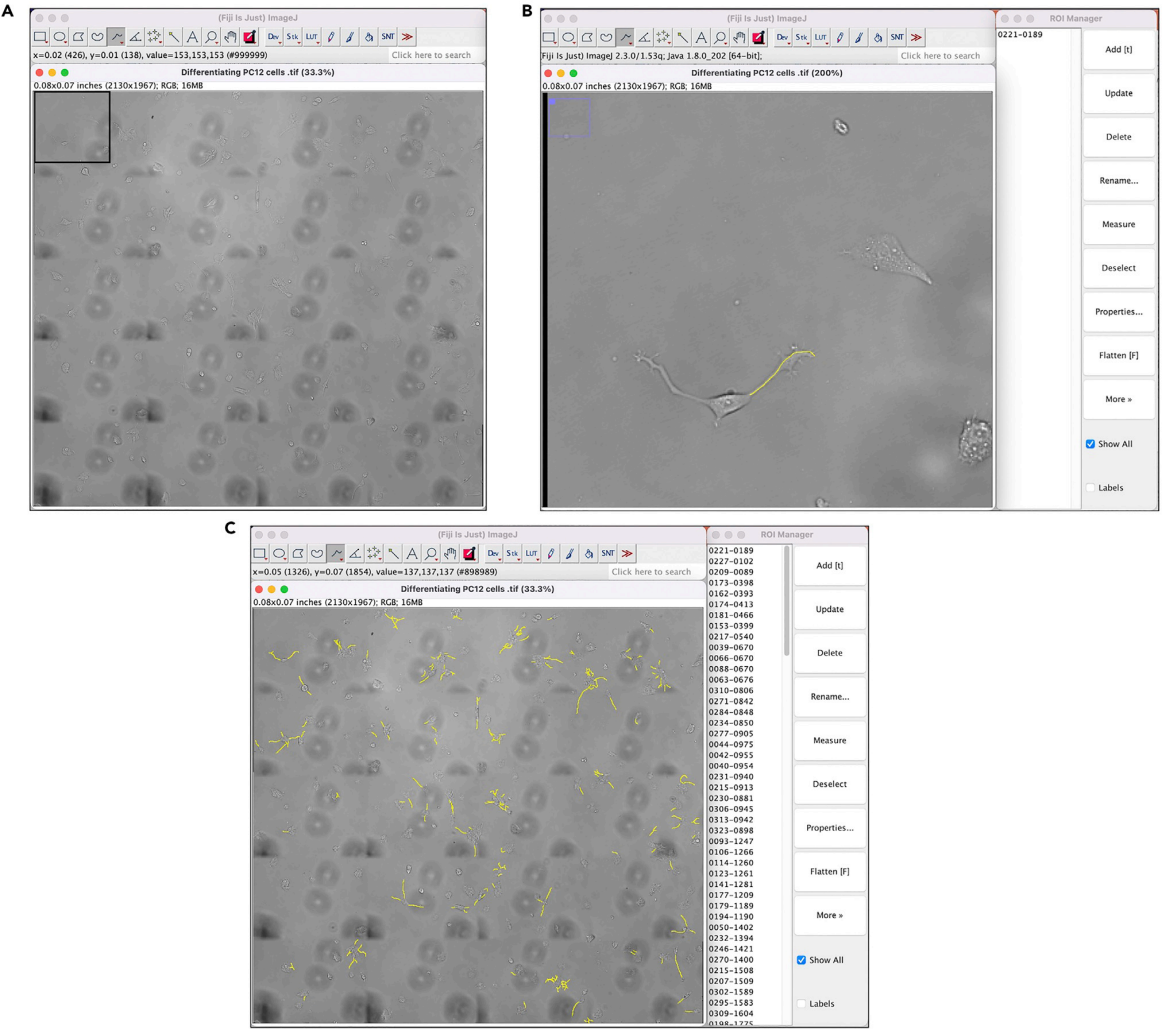
Workflow for neurite density quantification using Fiji (A) Tiled image opened in Fiji. (B) Zoom of black box in (A) showing a single neurite traced (yellow) and added to the ROI manager. (C) All neurites in the tiled image traced (yellow) and added to the ROI manager.
2. Zoom in on the image until individual cells and neurites are easily visible.
3. Using the freehand line tool, trace the first visible neurite and add it to the ROI manager (Figure 8B).
  a. Check the box next to “Show All” in the ROI manager so traces remain visible on the image.
4. Repeat step 3 to trace every neurite in the image (Figure 8C).
  a. To ensure no region of the imaged is skipped, we recommend analyzing from top to bottom, left to right.
5. After tracing every neurite, save the ROIs.
6. Click “Measure” in the ROI manager to measure the length (in inches) of each neurite. Save the measurements.
7. Open the measurements in Microsoft Excel (or other spreadsheet program) and sum the individual neurite lengths to calculate the total neurite length in the image.
8. Convert the total neurite length from inches to μm with the following conversion:
  a. 1 inch = 25,400 μm.
9. The total neurite density (μm/mm^2^) is the value calculated in step 8 / 3.3mm^2^ (the area of 10% of a 96-well plate (the region imaged)).

## Limitations

This protocol does not yield 100% differentiation of PC12 cells. The percent of cells that are differentiated varies between clonal variants and may be impacted by the distribution of cells in the well while plating or the propensity of cells to clump and proliferate. Performing single cell analyses such as immunocytochemistry avoids this limitation by allowing the researcher to select the differentiated cells for analysis. However, bulk analyses such as western blot cannot exclude undifferentiated cells and are therefore not completely accurate measures of the differentiated population. To analyze differentiated cells through western blot, normalize the protein(s) of interest to β-III-Tubulin expression for each sample, which will account for differences in the percent of cells that are differentiated between cell lines/wells.

## Troubleshooting

### Problem 1

**Figure 9:**
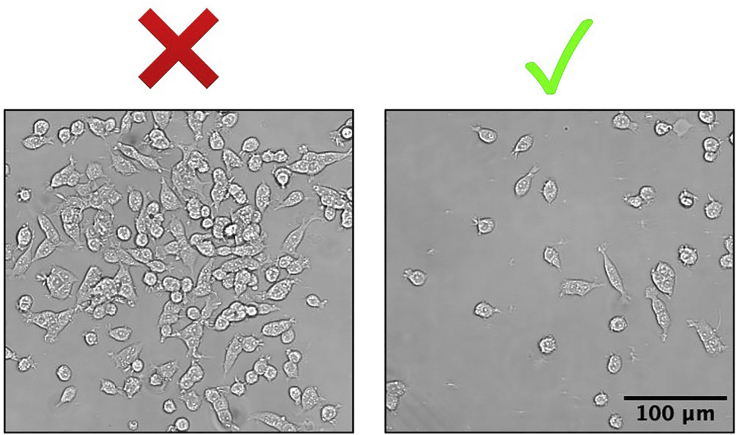
Poor and proper dispersion of PC12 cells in a well An example of poor (left) and proper (right) dispersion of PC12 cells after plating. Cells should be plated as single cells and evenly distributed throughout the well. Images are cropped for clarity.

PC12 cells are clumped together and not evenly distributed throughout the well (Plating PC12 cells, steps 6–21) (Figure 9).

### Potential solution

Cells might be clumped together in the well due to failure to create a single cell suspension before plating or poor plating technique.

• Creating a single cell suspension:
  ○ To create a single cell suspension, it is crucial to follow steps 7–14 exactly, especially passing cells through a 26-gauge syringe needle. Verify that cells are not in clumps when counting on the hemocytometer (step 15). To prevent cells from re-pelleting and clumping throughout the plating steps, periodically pipette the PC12 cell suspension and diluted PC12 cell suspension up and down.
• Poor plating technique:
  ○ Hold the pipette completely vertical and aim for the center of the well when plating cells to allow an even distribution of cells throughout the well. Keep the plate flat while plating and do not lift or tilt the plate for 5–10 min after plating to allow cells to settle in the well (generally, enough time has elapsed after filling surrounding wells with DPBS) (Figure 2C). Hold the plate horizontal and do not tilt it when putting it in the incubator. Do not look at the cells under the microscope until 2 h have elapsed to prevent disrupting the cells before attachment to the wells.

### Problem 2

Autofocus fails on the EVOS M7000 (step 26d).

### Potential solution

Autofocus can fail if cells are sparsely plated, as needed in this differentiation protocol. Changing the field acquisition order so that a different field of view is imaged first may resolve the issue, but it is not guaranteed. If autofocus continues to fail, manually focus each well prior to imaging.

### Problem 3

Cells begin to clump and/or the culture is overtaken by proliferation of undifferentiated cells when differentiating past a neurite density of ∼1,500 μm/mm^2^ (step 27).

### Potential solution

Treat cells with cytosine-beta-D-arabinofuranose hydrochloride (AraC) for 48 h at a final concentration of 1 μM starting on the day cells reach the target neurite density. AraC interferes with DNA synthesis and therefore results in cell death of dividing cells without affecting neuronal cell survival or neurite outgrowth.^4^ We have verified that AraC treatment eliminates proliferating cells and reduces clumping of neurite-bearing cells with undifferentiated cells (Figure 4).

***Note:*** The plating density may need to be increased to 1.5 × 10^4^ or 2.0 × 10^4^ cells/cm^2^ to account for the decrease in cell number from AraC-induced cell death.

### Problem 4

Low protein concentration in cell lysates (western blot analysis steps 36 and 40).

### Potential solution

Completely aspirate all DPBS prior to adding lysis buffer to the wells to avoid diluting the cell lysates. It may also be necessary to decrease the volume of lysis buffer added to each well. Use Figure 5 as a guide to estimate starting lysis buffer volumes based on cell confluency and adjust as necessary.

### Problem 5

Abundance of neurite/cell loss after performing immunocytochemistry (immunocytochemistry steps 57 and 59).

### Potential solution

Cell loss during the staining process may be due to improper coating of the wells (see coating wells). Verify that PDL is the proper molecular weight of 50,000–150,000 Daltons (best for use in neuronal culture), laminin aliquots are less than 6 months old, and laminin is thawed and used on ice. During the staining process, remove all liquid gently by pipette by placing the pipette against the edge of the well. Be very gentle when dispelling liquid down the side of the well. Washes may be performed without the use of the orbital shaker to prevent potential cell detachment, but this may result in slightly increased background signals.

## Resource availability

### Lead contact

Further information and requests for resources and reagents should be directed to and will be fulfilled by the lead contact [Diane E. Merry] (diane.merry@jefferson.edu).

### Materials availability

This study did not generate new unique reagents.

## Data Availability

This study did not generate any datasets or code.
